# Standardized ileal digestible lysine (protein) intake by primiparous sows should be increased in late gestation to maximize whole-body nitrogen retention, piglet birth weight, and subsequent milk yield

**DOI:** 10.1093/jas/skaf271

**Published:** 2025-08-18

**Authors:** Vanessa Kloostra, Chantal Farmer, Lee-Anne Huber

**Affiliations:** Department of Animal Biosciences, University of Guelph, Guelph, ON, CanadaN1G 2W1; Agriculture and Agri-Food Canada, Sherbrooke Research and Development Centre, Sherbrooke, QC, CanadaJ1M 0C8; Department of Animal Biosciences, University of Guelph, Guelph, ON, CanadaN1G 2W1

**Keywords:** late gestation, piglet birth weight, primiparous sows, standardized ileal digestible lysine intake, subsequent milk yield

## Abstract

One hundred fifty-two gestating primiparous sows were used to determine the standardized ileal digestible (**SID**) Lys (protein) intake in late gestation necessary to maximize whole-body N retention, piglet birth weight, and subsequent milk yield. On day 90 of gestation, primiparous sows were assigned to 1 of the 7 isoenergetic diets with equally spaced and increasing SID Lys (protein) contents using primarily soybean meal to supply additional Lys [70% to 160% of NRC-NRC-(2012) estimated requirements; 13.3 to 30.5 g SID Lys per day; *n* = ~ 21]. An N-balance was completed between days 105 and 108 of gestation. After farrowing, all sows received a standard lactation diet until weaning (day 20 ± 1). Using the Bayesian information criteria to assess best fit, the linear broken-line model was selected to determine optimal SID Lys (protein) intakes. Sow BW gain in late gestation increased, and BW loss in the subsequent lactation tended to increase with increasing SID Lys (protein) intake in late gestation (linear; *P *< 0.001 and *P *= 0.086, respectively). The gain in backfat depth during late gestation tended to decrease with increasing SID Lys (protein) intake (linear; *P *= 0.067), but backfat depth loss during the subsequent lactation was not influenced by SID Lys (protein) intake in late gestation. The N intake, excretion, and whole-body retention (N intake—N output in urine and feces) increased with increasing SID Lys (protein) intake (linear; *P *< 0.0001). Whole-body N retention was maximized at an SID Lys intake of 22.0 g/d during late gestation (115% of NRC-estimated requirements). Litter size and the number of stillborns per litter were not affected by dietary treatment, but piglet birthweight increased and then decreased with increasing SID Lys (protein) intake (quadratic; *P *< 0.01) and was maximized at an SID Lys intake of 22.0 g/d during late gestation. Estimated milk yield in the subsequent lactation increased (linear; *P *< 0.05) and litter growth rate tended to increase (linear; *P *= 0.057) with SID Lys (protein) intake in late gestation. Estimated milk yield was maximized at an SID Lys intake of 22.7 g/d during late gestation (119% of NRC-estimated requirements). Therefore, SID Lys intake in late gestating primiparous sows should be increased by 15% above current recommendations to maximize whole-body N retention in late gestation and piglet birth weight, and by 19% to maximize milk yield in the subsequent lactation.

## Teaser

Increasing the standardized ileal digestible Lys intake to 22.7 g/d between day 90 of gestation and farrowing maximized late-gestation whole-body nitrogen retention, piglet birth weight, and estimated milk yield in the subsequent lactation for primiparous sows.

## Introduction

Recent genetic advances in litter size have resulted in a greater incidence of low birthweight piglets (König et al., 2021). Moreover, sow milk production has not increased to the same degree as litter size, thus growth rates and preweaning mortality of piglets have been negatively affected (Milligan et al., 2002; Chisoro et al., 2023). In late gestation, protein deposition in both the fetal and mammary pools increases exponentially, necessitating increased amino acid (**AA**) intakes by the primiparous sow to maximize mammary development (Farmer et al. 2022) and piglet body weight at birth (Rehfeldt et al., 2011; Farmer et al. 2022), and to limit mobilization of maternal protein pools prior to farrowing (McPherson et al., 2004; Ji et al., 2006; Dourmad et al., 2008).

Previous work has shown that increasing Lys intake above the currently perceived requirements for late-gestating primiparous sows improved piglet BW at birth (Heo et al., 2008) and piglet growth rates (milk production) in the ensuing lactation (Jang et al., 2014). It was postulated that the improvement in subsequent milk production was due to greater mammary development in late gestation. Indeed, Farmer et al. (2022) found that feeding standardized ileal digestible (**SID**) Lys (protein) 40% above the NRC- (2012) estimated requirements in late-gestating primiparous sows increased mammary parenchymal mass by 44% and fetal piglet body weight by 5% on day 110 of gestation (versus providing SID Lys at 100% of estimated requirements). In the aforementioned study, primiparous sows were slaughtered at the end of gestation; thus, subsequent milk production could not be assessed. Moreover, only 2 dietary Lys levels were used, meaning optimal feeding levels could not be established, and precise feeding recommendations could not be made. Thus, it was expected that as SID Lys intake increased above current requirements, mammary development and subsequent milk production would also increase, reflecting that current requirement estimates were established primarily based on piglet birth weight and whole-body protein deposition, without considering subsequent milk production as an indicator of AA adequacy in late gestation.

The objective of the current study was to determine the intake of SID Lys (protein) in late gestating primiparous sows that maximized whole-body protein deposition, as well as piglet birth weight and milk production in the following lactation period.

## Materials and Methods

The experimental protocol was approved by the University of Guelph Animal Care Committee and followed the Canadian Council on Animal Care guidelines (CCAC, 2009; Animal utilization protocol #4889). The study was conducted at the Ontario Swine Research Center (Elora, ON, Canada).

### Animals and housing

One hundred fifty-two primiparous sows (PIC Camborough; Pig Improvement Company, Winnipeg, MB, Canada) were recruited to the study on day 89 of gestation (initial BW of 202.1 kg ± 1.1) over 7 breeding batches (blocks). Prior to the study and outside nitrogen balance periods, primiparous sows were housed in static groups of approximately 25 animals in pens equipped with an automatic sow feeding system with feed blending capabilities (Gestal 3G2; JYGA Technologies, St-Lambert-de-Lauzon, QC, Canada) and supplied by 4 feed lines. During the nitrogen (**N**) balance periods (i.e., between days 104 and 110 of gestation), a subset of primiparous sows was housed in gestation stalls. On day 110 of gestation, all primiparous sows were moved to farrowing crates equipped with electronic feeders (Gestal Quattro; JYGA Technologies, St-Lambert-de-Lauzon, QC, Canada). In both gestation and lactation, each feeder head was calibrated weekly. Primiparous sows were induced on day 115 of gestation using 2 mL of Planate (Merck & Co., Inc., Kirkland, QC; 175 µg cloprostenol) if farrowing had not already occurred. Litters were standardized, based on piglet availability and regardless of maternal dietary treatment, to achieve a litter size of 13 ± 1. Piglets were processed according to farm protocol (ear notching and teeth clipping within 24 h of birth and iron injection, tail docking, and surgical castration of male piglets 4 d after birth). Creep feed was not provided, so that piglet BW gain was reflective of sow milk production.

### Dietary treatments and feeding

From breeding until day 89 of gestation, all primiparous sows received 2.20 kg of a standard gestation diet (2,382 kcal/kg net energy, 12.28% crude protein, 0.55% SID Lys). On day 89 of gestation, primiparous sows were assigned to 1 of the 7 dietary treatments to ensure comparable initial BW across treatments and received 2.65 kg per day of the assigned diet until farrowing. Three isoenergetic basal diets were formulated to contain 0.50%, 0.83%, or 1.15% SID Lys, which represented 70%, 115%, and 160% of NRC- (2012) estimated SID Lys requirements for primiparous sows between days 90 and 114 of gestation using the following inputs in the gestating sow model: sow BW at breeding of 150 kg, 114-d gestation, anticipated litter size of 15.5, and an anticipated piglet birth weight of 1.4 kg (Table 1), which corresponded to target daily SID Lys intakes between 13.3 and 30.1 g/d during the late gestation feeding period. Soybean meal and crystalline Lys were used to increase SID Lys contents. All other AA met or exceeded estimated requirements and met or exceeded the recommended AA: Lys ratios according to the NRC (2012) using crystalline AA, as needed, and to ensure Lys remained the first-limiting AA. Corn, full-fat soybeans, soybean hulls, and soybean oil were also used to balance energy and fiber contents among the basal diets. Two of the basal gestation diets were blended in different proportions by the electronic sow feeders to achieve 4 additional intermediate SID Lys contents [i.e., the 70 and 115 % diets were blended to achieve 85% and 100% and the 115% and 160% diets were blended to achieve 130% and 145% of the NRC- (2012) estimated SID Lys requirements]. Titanium dioxide was included as an indigestible marker to determine apparent total tract nitrogen digestibility.

**Table 1.** Ingredient and nutrient composition of the experimental gestation diets (as-fed)

	Basal gestation diets^1^		
	70	115	160
Ingredient, %			
Corn	53.51	46.87	40.30
Wheat	20.00	20.00	20.00
Soybean meal	8.50	24.00	30.00
Full fat soybeans	5.00	3.00	4.00
Soybean hulls	7.30	1.00	--
Soybean oil	2.01	1.75	1.93
Limestone	1.17	1.23	1.23
Calcium phosphate	1.34	1.15	1.05
Vitamin and mineral premix ^2^	0.60	0.60	0.60
Sodium chloride	0.30	0.30	0.30
DL-Met	0.05	--	0.16
L-Thr	0.11	--	0.13
L-Trp	0.01	--	--
L-Lys-HCl	--	--	0.20
Titanium dioxide	0.10	0.10	0.10
Total	100.00	100.00	100.00
Calculated nutrient contents ^3^			
Net energy, kcal/kg	2,500	2,500	2,500
Crude protein, %	13.40	18.73	21.70
SID Lys, %	0.50	0.83	1.15
SID Ile, %	0.43	0.67	0.78
SID Met, %	0.25	0.27	0.45
SID Met + Cys, %	0.47	0.55	0.76
SID Thr, %	0.49	0.57	0.79
SID Trp, %	0.14	0.20	0.24
SID Val, %	0.50	0.74	0.84
SID Arg, %	0.68	1.09	1.28
SID His, %	0.30	0.44	0.50
SID Leu, %	0.98	1.34	1.49
SID Phe, %	0.54	0.80	0.92
SID Phe + Tyr, %	0.90	1.32	1.51
STTD P, % ^4^	0.38	0.38	0.38
Total Ca, %	0.88	0.88	0.88
Fermentable fiber, %	10.52	11.26	12.12

^1^Two of the basal gestation diets were blended in different proportions by the electronic sow feeders to achieve 4 additional intermediate SID Lys contents [i.e., the 70% and 115 % diets were blended to achieve 85% and 100% and the 115% and 160% diets were blended to achieve 130% and 145% of the NRC- (2012) estimated SID Lys requirements].

^2^Provided the following amounts of vitamins and trace minerals per kg of premix: vitamin A, 2,000 KIU; vitamin D, 200 KIU; vitamin E, 8,000 IU; vitamin K, 500 mg; thiamin, 300 mg; riboflavin, 1,000 mg; niacin, 5,000 mg; pantothenic acid, 3,000 mg; pyridoxine, 300 mg; choline, 100,000 mg; folacin, 400 mg; biotin, 40 mg; vitamin B12, 5,000 µg; calcium, 22.67% as CaCO_3_; manganese, 4,000 mg as Mn SO_4_·H_2_O; zinc, 21,000 mg as ZnSO_4_; iron, 20,000 mg as FeSO_4_; copper, 3,000 mg as CuSO_4_; selenium, 60 mg as Na_2_SeO_3_; iodine, 100 mg as C_2_H_10_I_2_N_2_ (Grand Valley Fortifiers, Cambridge, ON, Canada).

^3^Based on digestible nutrient and net energy contents of feed ingredients according to the NRC (2012).

^4^Standardized total tract digestible.

While housed in groups, primiparous sows received the blends via the electronic sow feeder, but during nitrogen balance periods and between day 110 of gestation and farrowing, the dietary treatments were blended and dispensed manually in 2 equal meals, with any feed refusals recorded on a daily basis. After farrowing, all primiparous sows received a standard lactation diet via the electronic sow feeders in a step-up program with ad libitum feed access achieved 7 d after farrowing (Table 2). Dispensed feed was recorded daily using the electronic feeding system and feed refusals were measured manually twice per week to estimate average daily feed intake. Sows (and piglets) received ad libitum access to water throughout the study.

**Table 2.** Analyzed nutrient composition (%) of the experimental basal gestation diets and common lactation diet (as-fed)

	Basal gestation diets^1^			Lactation diet
	70	115	160	
Crude protein^2^	14.13	18.81	21.16	-
Lys^3^	0.64 (0.62)	0.97 (0.97)	1.20 (1.31)	1.22
Ile	0.64 (0.51)	0.83 (0.76)	0.98 (0.89)	0.80
Met	0.32 (0.28)	0.34 (0.30)	0.49 (0.49)	0.68
Met + Cys	0.67 (0.55)	0.68 (0.64)	0.85 (0.87)	1.08
Thr	0.63 (0.59)	0.58 (0.69)	0.89 (0.93)	0.77
Val	0.73 (0.61)	0.91 (0.86)	1.05 (0.99)	0.97
Arg	0.83 (0.76)	1.12 (1.18)	1.38 (1.39)	1.17
His	0.42 (0.36)	0.51 (0.50)	0.60 (0.57)	0.46
Leu	1.38 (1.15)	1.58 (1.54)	1.91 (1.72)	1.54
Phe	0.82 (0.64)	1.02 (0.92)	1.27 (1.05)	0.98

^1^Two of the basal gestation diets were blended in different proportions by the electronic sow feeders to achieve 4 additional intermediate SID Lys contents [i.e., the 70% and 115% diets were blended to achieve 85% and 100% and the 115% and 160% diets were blended to achieve 130% and 145% of the NRC- (2012) estimated SID Lys requirements].

^2^Analyzed values for composite sample of 5 batches per diet.

^3^Calculated values are shown in parentheses (%).

### Performance measurements, nitrogen balance procedure, and blood and milk sampling

On days 89 and 110 of gestation, within 24 h of farrowing, and at weaning (day 20 ± 1), sows were weighed. Backfat depth was also measured at the P2 position on days 89 and 110 of gestation and at weaning using an ultrasound (Honda Electric Co., Ltd., Toyohashi, Aichi, Japan). After farrowing, the number of piglets born alive, stillborn, and mummified were recorded. Body weights were recorded within 24 h of birth for all live and stillborn piglets. Piglet BW were also collected on days 7, 14, and at weaning, and mortalities were recorded with dates and BW to calculate litter ADG and estimated milk yield.

On day 105 of gestation, urinary catheters (BARDEX I.C., 2-way, Specialty, Tiemann Model, 30cc balloon, 18FR, Bard Medical, Covington, GA) were inserted into the bladder of a subset of sows (*n* = 10 or 11) to allow for total urine collection. The balloon was filled with 35 mL of saline solution to hold the catheter in place, and the catheter was connected to a sealed collection vessel with polyvinyl tubing. Sulfuric acid was used to keep the pH in the container less than 3. Following each successful 24-h collection, subsamples of urine (5% of total urine weight) were collected and pooled within sow and stored at 4 ^°^C. Urine collection continued until 3 successful 24-h collection periods were achieved or until day 109 of gestation, whichever occurred first. Thereafter, urinary catheters were removed. At the end of the nitrogen balance period, the pooled urine samples from each sow were mixed, subsampled, and stored at −20 °C until further analysis. Fecal samples were collected each day during the nitrogen balance period, pooled for each sow, and stored at −20 °C until further analysis.

On day 110 of gestation, suborbital sinus blood samples were collected from a second subset of sows (*n* = 10 or 11). Animals were fasted for at least 16 h prior to collecting 10 mL of blood into heparinized tubes (BD Vacutainer; Franklin Lakes, NJ, USA). Within 20 min of collection, blood samples were centrifuged for 12 min at 1,800 × *g* at 4^o^C. Plasma was recovered and stored in microcentrifuge tubes at −20 °C until further analysis.

Upon farrowing, a total of 150 mL of colostrum was collected from 2 teats (the first or second gland and the last or second-last gland) from a subset of sows (*n* = 13 to 17) approximately 24 h after birth of the first piglet with the precise time between birth of the first piglet to colostrum collection later confirmed by video recordings. On day 18 of lactation, a total of 150 mL of milk was collected from 2 teats (the first or second gland and the last or second-last gland) from an additional subset of sows (*n* = 13 to 15) after piglets were removed from the sow and placed in an adjacent farrowing crate for 1 h to allow for milk accumulation. Prior to the collection of colostrum and milk, sows received 1.5 mL oxytocin (Oxyto-Sure, Vetoquinol, Cambridge, ON; 20 USP/mL) via intramuscular injection. Samples were pooled for each sow and stored at −20 °C until further analysis.

### Sample analyses

Samples were collected biweekly from 5 batches of experimental diets, and a representative composite sample of the 5 batches was then used for chemical analysis. Representative subsamples of the lactation diet were also collected weekly throughout the trial. Fecal samples were freeze-dried, and then feed and freeze-dried feces were finely ground. Feed (in quadruplicate) and feces (in duplicate) were analyzed for dry matter at 135 °C for 2 h (AOAC, 2005; method 930.15) and analyzed for ash contents at 600 °C for 12 h in a muffle furnace (AOAC, 2005; method 942.05). Titanium contents in feed and fecal samples were determined as described by Crosbie et al. (2020).

Colostrum and milk samples were vortexed and then freeze-dried to determine dry matter content. Crude fat content of the dried samples was then determined by high-temperature solvent extraction (Ankom, XT29 Fat Analyzer, Macedon, NY; AOCS, 2017; Official Procedure Am 5-04). Colostrum and milk lactose concentrations were determined using a colorimetric enzymatic method (Neogen, Lansing, MI, USA) with 30 µL of galactosidase and a final incubation time of 60 min, with intra- and interassay CVs of 2.15% and 2.05%, respectively. The N content was determined for feed, urine, freeze-dried feces, colostrum, and milk using combustion analysis (LECO-FP 828 analyzer; LECO instruments ltd., Mississauga, ON, Canada). Crude protein contents of feed, urine, and feces were calculated as N × 6.25, and crude protein contents of colostrum and milk were calculated as N × 6.38.

Amino acid contents of feed and plasma were analyzed using ultra-performance liquid chromatography (Waters Corporation, Milford, MA) as described by Banton et al. (2021). The feed samples underwent oxidized hydrolysis before analysis. Derivatization was completed using an AccQ-Tag Ultra derivatization kit (Waters Corporation, Milford, MA). The obtained amino acid peak areas were compared with known standards, and the data were analyzed using Waters Empower 2 Software (Waters Corporation, Milford, MA).

### Calculations and statistical analyses

The N intake during the nitrogen balance period was calculated by multiplying the analyzed N content of the diets and the daily feed intakes. Fecal N excretion was calculated using N intake and apparent total tract digestibility of N, while N absorbed and N retained were calculated by subtracting daily N output in feces and N output in feces and urine, respectively, from N intake (Miller et al., 2019). Milk yield was estimated according to the NRC (2012) based on litter size, piglet ADG, and a standard lactation curve (NRC, 2012; Equations 8-71 and 8-72).

The Proc GLIMMIX function of SAS was used for all statistical analyses. Sow (or litter) was used as the experimental unit. Dietary treatment in gestation was a fixed effect, while block was considered a random effect. Plasma AA concentrations were log-transformed for analysis and then back-transformed for data presentation. The covariate of time between birth of the first piglet, as determined via the video recordings, and colostrum sample collection was used for colostrum composition analyses, and the covariate of litter size was used for both piglet and litter birth weights. Linear and quadratic contrast statements were used to assess the effects of increasing SID Lys intake in late gestation. A probability (***P***) value of less than 0.05 was considered significant, while 0.05 ≤ *P *≤ 0.10 was considered a trend. Breakpoint analyses were conducted according to Gonçalves et al. (2016), using quadratic polynomial, broken-line linear (**BLL**), and broken-line quadratic models for whole-body N retention, apparent efficiency of N retention, piglet birthweight, and estimated milk yield in the subsequent lactation period. The Bayesian information criterion was used to assess the best fit.

## Results

The analyzed Lys contents in feed were comparable to the calculated nutrient contents for all basal diets fed in late gestation (Table 2). Estimated daily SID Lys intake ranged between 13.7 and 27.6 g/d during the late gestation feeding period (Table 3). For Ile, Met, Val, His, Leu, and Phe, the analyzed contents were 13% to 22% greater than calculated values. Twenty-four animals were removed from the study (7, 2, 3, 3, 1, 4, and 4 sows from the 70%, 85%, 100%, 115%, 130%, 145%, and 160% treatments, respectively) for various reasons including abortion, lameness, farrowing difficulties, and savaging of piglets. Data from sows that were removed before or during lactation were not included in statistical analyses of lactation variables.

**Table 3.** Performance of primiparous sows during gestation and lactation when fed diet blends that provided increasing and equally spaced standardized ileal digestible Lys

	Diet^1^							SEM^2^	*P*-value^3^	
	70	85	100	115	130	145	160		Linear	Quadratic
Number of sows gestation	22	21	23	22	21	21	22			
Number of sows lactation^4^	15	19	20	19	20	17	18			
Estimated SID Lys intake, g/d^5^	13.7	16.4	19.2	22.0	24.0	25.9	27.6			
Body weight, kg										
Day 89	202.9	201.3	202.1	203.4	202.6	203.6	202.0	3.1	0.883	0.888
Day 110	222.6	225.0	227.1	227.5	228.0	229.3	226.9	3.6	0.142	0.284
Post-farrow	200.3	202.6	204.0	204.2	200.6	206.4	205.9	3.0	0.168	0.754
Weaning^6^	185.0	184.7	186.7	185.4	183.1	187.3	185.6	3.7	0.874	0.925
Body weight change, kg										
Days 89 to 110	19.6	23.6	25.0	24.0	25.3	25.7	25.0	1.1	<0.001	0.010
Farrowing to weaning	−15.6	−18.0	−17.1	−18.7	−18.0	−19.1	−20.6	2.8	0.086	0.941
Backfat depth, mm										
Day 89	12.3	13.0	12.4	12.2	11.7	12.3	12.9	0.7	0.883	0.273
Day 110	13.2	13.7	13.2	12.2	12.4	12.7	13.2	0.7	0.252	0.166
Weaning	9.6	9.8	9.5	9.0	8.7	9.4	10.0	0.5	0.927	0.074
Backfat depth change, mm										
Days 89 to 110	0.9	0.7	0.8	0.0	0.7	0.4	0.3	0.3	0.067	0.529
Day 110 to weaning	−3.5	−3.6	−3.4	−3.4	−3.7	−3.4	−3.4	0.4	0.772	0.921

^1^Standardized ileal digestible Lys level in the diet as a percent of estimated requirements for late gestating primiparous sows according to the NRC (2012).

^2^Maximum value for the standard error of the mean.

^3^Probability values for linear and quadratic contrasts.

^4^Twenty-four animals were removed from the study (7, 2, 3, 3, 1, 4, and 4 sows from the 70%, 85%, 100%, 115%, 130%, 145%, and 160% treatments, respectively) for various reasons including abortion, lameness, farrowing difficulties, and savaging of piglets. Data from sows that were removed before or during lactation were not included in statistical analyses of lactation outcomes.

^5^Calculated using total analyzed Lys content of the diet, Lys standardized ileal digestibility coefficient (NRC, 2012), and observed feed intake.

^6^Weaning occurred on day 20 ± 1 of lactation.

Gestation diet did not influence the BW of sows at any time point; however, BW gain in late gestation increased (linear and quadratic; *P *< 0.001 and *P *< 0.05, respectively) and BW loss in lactation tended to increase with increasing SID Lys intake in late gestation (linear; *P* = 0.086; Table 3). Backfat depth did not differ between treatments on days 89 or 110 of gestation, but by weaning, it tended to decrease and then increase with increasing SID Lys intake during late gestation (quadratic; *P = *0.074). During late gestation, backfat depth gain tended to decrease with increasing SID Lys intake (linear; *P* = 0.067) but no treatment differences were observed for backfat depth change during lactation.

**Figure 1.**
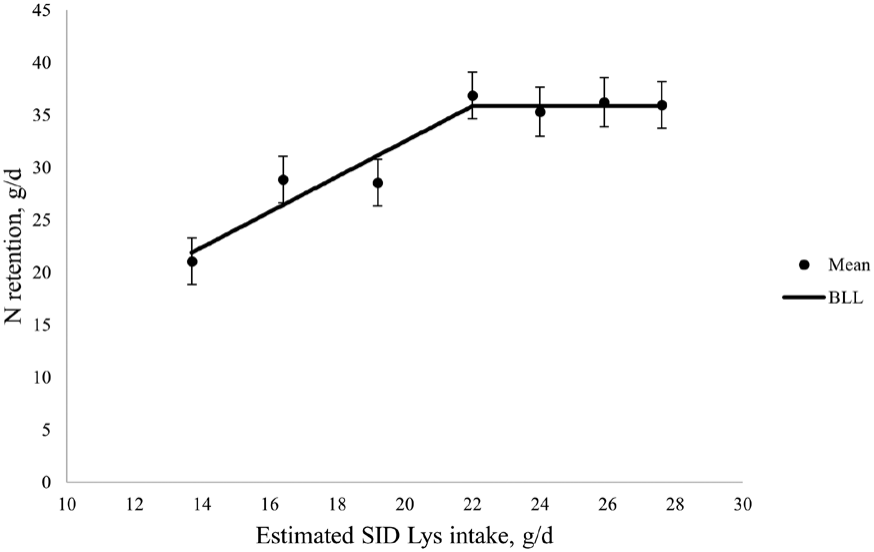
The effect of increasing standardized ileal digestible (SID) Lys intake in late gestation on whole-body nitrogen retention between days 105 and 108 of gestation. Nitrogen retention increased until a breakpoint at 22.0 ± 1.2 g SID Lys/d and reached a plateau at 35.9 g/d according to the broken-line linear (BLL) model. For Xi < 22.0, N retention, g/d = −1.1812 + 1.6841 × (Xi), where Xi is the SID Lys intake in g/d, for an individual sow, i. Closed circles represent the least squares means, and the error bars indicate SEM for the least squares mean.

**Figure 2.**
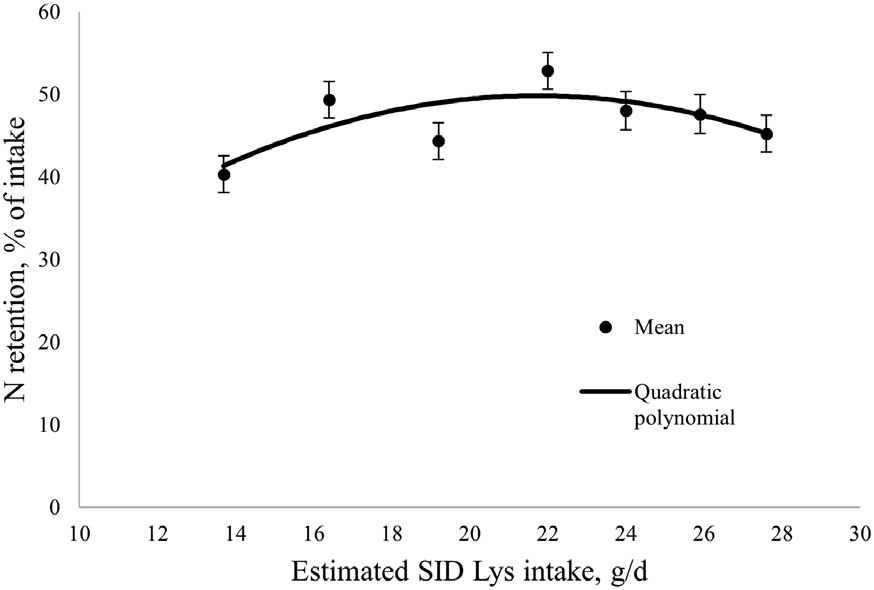
The effect of increasing standardized ileal digestible (SID) Lys intake in late gestation on apparent nitrogen retention efficiency between days 105 and 108 of gestation. Nitrogen retention efficiency increased then decreased with increasing SID Lys intake, being maximized at 21.7 ± 13.4 g SID Lys/d, which was at a level of 49.8% according to the quadratic polynomial model. N retention efficiency, % = -0.132 × (Xi)^2 + 5.7336 × (Xi) −12.4344, where Xi is the SID Lys intake in g/d, for an individual sow, i. Closed circles represent the least squares means, and the error bars indicate SEM for the least squares mean.

During the nitrogen balance between days 105 and 108 of gestation, N intake, total N excretion (in both urine and feces), N absorbed (N intake—N excreted in feces), N excretion in urine, and N retention, all increased with increasing SID Lys intake (linear; *P* < 0.001; and also quadratic for N intake, fecal N excretion, N absorbed, and N retained; *P *< 0.05; Table 4). The apparent N utilization efficiency (N retention as a percent of N intake) increased then decreased with increasing SID Lys intake (quadratic; *P* < 0.05). The BLL model had the best fit for N retention, whereby N retention increased until a breakpoint at 22.0 ± 1.2 g SID Lys/d and reached a plateau at 35.87 g/d, which corresponded to 115% of the current NRC- (2012) estimated requirements (Figure 1). The quadratic polynomial model had the best fit for apparent N utilization efficiency, whereby apparent N utilization efficiency was maximized at 21.7 ± 13.4 g/d SID Lys, and peaked at 49.83%, which corresponded to 114% of current NRC- (2012) requirements (Figure 2).

**Table 4.** Nitrogen utilization of primiparous sows between days 105 and 108 of gestation when fed diet blends that provided increasing and equally spaced standardized ileal digestible Lys

	Diet^1^							SEM^2^	*P*-value^3^	
	70	85	100	115	130	145	160		Linear	Quadratic
Number of sows	11	11	11	11	10	10	11			
N intake, g/d	52.5	58.5	64.5	69.7	73.7	76.1	79.5	0.9	< 0.001	< 0.001
Total N excretion, g/d	31.3	29.6	35.7	32.9	38.1	39.5	43.8	2.1	< 0.001	0.226
Fecal N excretion, g/d	10.5	9.0	9.2	8.7	9.0	8.4	9.1	0.9	0.026	0.035
Urine N excretion, g/d	20.9	20.3	27.1	23.5	27.9	30.9	34.3	1.8	< 0.001	0.326
N absorbed, g/d	42.0	49.5	55.3	61.0	64.7	67.7	70.4	1.6	< 0.001	< 0.001
N retention, g/d	21.1	28.9	28.6	36.9	35.3	36.2	36.0	2.3	< 0.001	0.004
N retention, % of intake	40.3	49.3	44.3	52.8	48.0	47.6	45.2	3.3	0.329	0.024

^1^Standardized ileal digestible Lys level in the diet as a percent of estimated requirements for late-gestating primiparous sows according to the NRC (2012).

^2^Maximum value for the standard error of the mean.

^3^Probability values for linear and quadratic contrasts.

On day 110 of gestation, plasma concentrations of His, Met, Phe, and Thr decreased (linear; *P* < 0.05; also quadratic for His, Met, and Thr; *P *< 0.05) and Arg tended to decrease (linear; *P* = 0.072) with increasing SID Lys intake (Table 5). Plasma concentrations of Ile and Lys increased (linear; *P* < 0.05) and total essential AA tended to decrease, then increase (quadratic; *P* = 0.054) with increasing SID Lys intake. Plasma concentrations of Ala, Asn, Asp, Glu, Gln, and total nonessential AA decreased (linear; *P* < 0.01), while Pro and Ser tended to decrease (linear; *P* = 0.075 and 0.052, respectively) with increasing SID Lys intake.

**Table 5.** Fasted plasma amino acid concentrations for primiparous sows on day 110 of gestation after receiving diet blends that provided increasing and equally spaced standardized ileal digestible Lys during late gestation

	Diet^1^							SEM^2^	*P*-value^3^	
	70	85	100	115	130	145	160		Linear	Quadratic
Number of sows	11	11	10	11	10	11	9			
EAA, μmol/L^4^										
Arg	154	145	133	154	135	139	134	7	0.072	0.814
His	121	108	100	102	97	109	100	6	0.016	0.018
Ile	119	113	116	136	131	135	131	8	0.022	0.652
Leu	217	183	190	198	187	193	194	12	0.461	0.246
Lys	88	100	95	96	114	136	124	17	0.002	0.726
Met	47	39	35	36	33	37	35	2	<0.001	0.001
Phe	99	85	84	92	86	81	85	5	0.048	0.340
Thr	206	142	130	138	130	146	148	10	< 0.001	< 0.001
Trp	70	59	63	59	58	66	60	4	0.359	0.160
Val	250	229	242	258	266	276	287	14	0.001	0.292
Total EAA	1,374	1,212	1,187	1,298	1,243	1,310	1,319	57	0.721	0.054
NEAA, μmol/L^5^										
Ala	585	535	541	549	433	518	453	39	0.002	0.843
Asn	47	40	39	42	38	38	36	2	<0.001	0.521
Asp	21	15	16	16	15	16	14	2	0.004	0.183
Cys	8	12	8	10	5	7	5	6	0.190	0.606
Glu^6^	245	200	202	203	165	189	166	13	<0.001	0.245
Gln	381	338	352	332	295	310	292	23	<0.001	0.650
Gly	1,034	1,014	998	982	986	1,025	948	67	0.488	0.972
Pro	309	261	272	287	243	280	254	19	0.075	0.459
Ser	166	143	143	153	144	149	132	10	0.052	0.917
Tyr	110	101	101	114	110	111	104	5	0.717	0.698
Total NEAA	2,953	2,679	2,700	2,736	2,418	2,678	2,407	135	0.004	0.724

^1^Standardized ileal digestible Lys level in the diet as a percent of estimated requirements for late-gestating primiparous sows according to the NRC (2012).

^2^Maximum value for the standard error of the mean.

^3^Probability values for linear and quadratic contrasts.

^4^EAA, essential amino acids; Arg is included as a conditionally EAA.

^5^NEAA, nonessential amino acids.

^6^Included citrulline.

**Figure 3.**
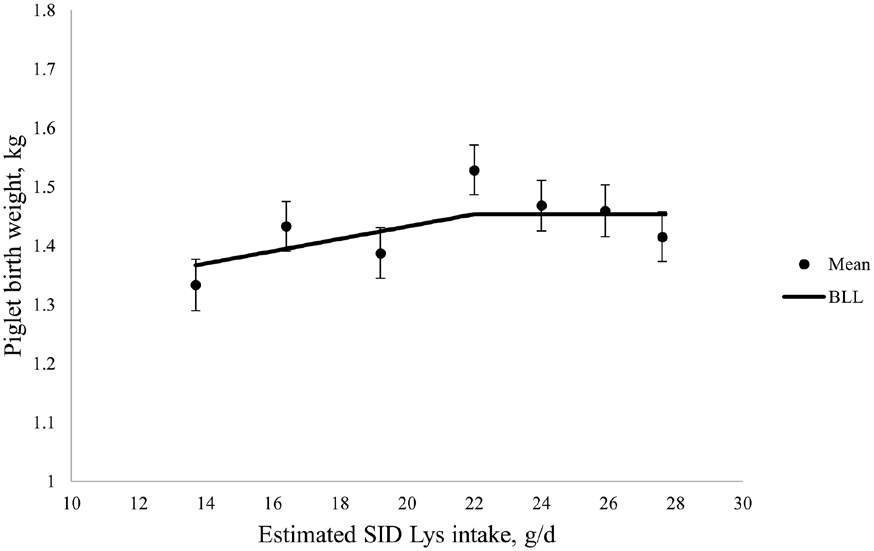
The effect of increasing standardized ileal digestible (SID) Lys intake between day 90 of gestation and farrowing on piglet birth weight. Piglet birth weight increased until a breakpoint at 22.0 ± 0.02 g SID Lys/d and reached a plateau at 1.45 kg according to the broken-line linear (BLL) model. For Xi < 22.0, piglet birth weight, kg = 1.2234 + 0.0105 × (Xi), where Xi is the SID Lys intake in g/d, for an individual sow, i. Closed circles represent the least squares means, and the error bars indicate SEM for the least squares mean.

The total number of piglets born, number of piglets born alive, variation in piglet birthweight, litter size after cross-fostering, and litter size at weaning were not impacted by SID Lys intake in late gestation (Table 6). Litter birthweight increased with increasing SID Lys intake in late gestation (linear; *P *< 0.01), and individual piglet birthweight increased then decreased (quadratic; *P* < 0.01) with increasing SID Lys intake in late gestation. The BLL model had the best fit for piglet birthweight, whereby piglet birthweight increased until a breakpoint at 22.0 ± 0.02 g SID Lys/d and reached a plateau at 1.45 kg, which corresponded to 115% of the current NRC- (2012) estimated requirements (Figure 3).

**Table 6.** Litter characteristics for primiparous sows after receiving diet blends that provided increasing and equally spaced standardized ileal digestible Lys during late gestation

	Diet^1^							SEM^2^	*P*-value^3^	
	70	85	100	115	130	145	160		Linear	Quadratic
Number of litters^4^	19	20	20	21	20	19	21			
Total born	13.5	14.9	15.5	14.7	15.6	14.5	15.1	0.9	0.299	0.180
Born alive	12.8	13.9	15.0	14.0	14.7	13.8	14.4	0.8	0.277	0.154
Stillborn	0.7	0.9	0.5	0.7	0.9	0.8	0.7	0.3	0.882	0.977
Mummified	0.4	0.5	0.5	0.5	0.6	0.7	0.6	0.2	0.202	0.961
Piglet birth weight, kg^5^	1.33	1.43	1.39	1.53	1.47	1.46	1.42	0.04	0.048	0.005
Litter birth weight, kg	19.4	20.4	19.7	21.5	21.3	21.6	20.8	0.6	0.008	0.105
CV piglet birth weights, %	19.4	20.1	20.4	18.3	19.3	20.9	18.8	1.5	0.878	0.933
Litter size^6^	13.3	12.8	13.8	13.3	13.3	13.3	13.7	0.4	0.186	0.830
Litter size at weaning^7^	11.8	12.2	12.3	12.4	12.6	12.7	12.5	0.5	0.101	0.401

^1^Standardized ileal digestible Lys level in the diet as a percent of estimated requirements for late gestating primiparous sows according to the NRC (2012).

^2^Maximum value for the standard error of the mean.

^3^Probability values for linear and quadratic contrasts.

^4^Prior to and during farrowing, 3, 1, 3, 1, 1, 2, 1 sows were removed from the 70%, 85%, 100%, 115%, 130%, 145%, and 160% treatments, respectively (vs Table 3) due to abortion, lameness, and farrowing difficulties.

^5^Prior to cross-fostering.

^6^After cross-fostering.

^7^Overall, 24 animals were removed from the study (7, 2, 3, 3, 1, 4, and 4 sows from the 70%, 85%, 100%, 115%, 130%, 145%, and 160% treatments, respectively) for various reasons including abortion, lameness, farrowing difficulties, and savaging of piglets. Data from sows that were removed before or during lactation were not included in statistical analyses of lactation outcomes, including litter size at weaning.

**Figure 4.**
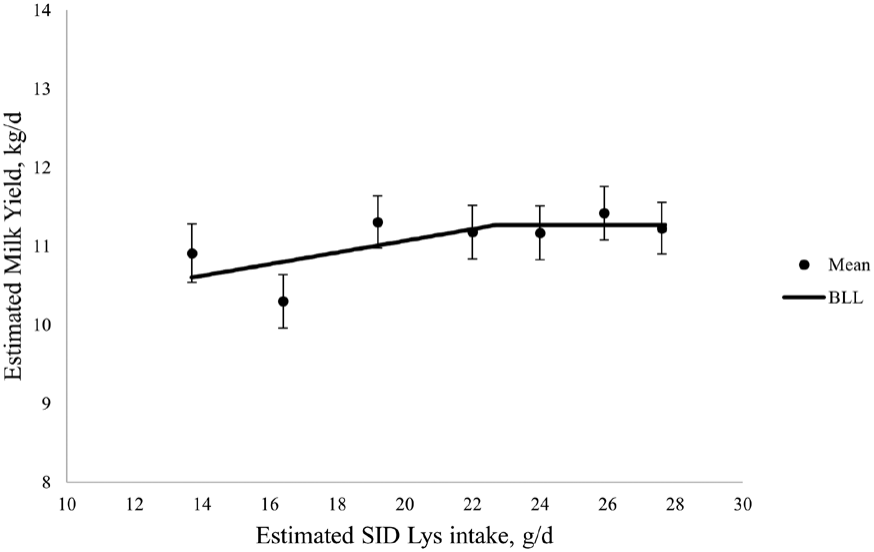
The effect of increasing standardized ileal digestible (SID) Lys intake between day 90 of gestation and farrowing on estimated subsequent milk yield. Estimated milk yield increased until a breakpoint at 22.7 ± 4.1 g SID Lys/d and reached a plateau at 11.3 kg/d according to the broken-line linear (BLL) model. For Xi < 22.7, estimated milk yield, kg/d = 9.586 + 0.0741 × (Xi), where Xi is the SID Lys intake in g/d, for an individual sow, i. Closed circles represent the least squares means, and the error bars indicate SEM for the least squares mean.

Total lactation sow feed intake tended to decrease then increase with increasing SID Lys intake in late gestation (quadratic; *P *= 0.067), but average daily feed intake over the entire lactation period was not influenced by SID Lys intake during gestation (Table 7). Estimated milk yield increased with increasing SID Lys intake in late gestation (linear; *P* < 0.05). Individual piglet BW (after litter standardization) did not differ at any time point, but litter ADG in weeks 2 and 3 and overall tended to increase with increasing SID Lys intake in late gestation (linear; *P *= 0.050, 0.068, and 0.057, respectively). The BLL model had the best fit for estimated milk yield, whereby milk yield increased until a breakpoint at 22.7 ± 4.1 g SID Lys/d and reached a plateau at 11.27 kg/d, which corresponded to 119% of current NRC- (2012) estimated requirements (Figure 4).

**Table 7.** Performance of primiparous sows and their piglets during lactation after receiving diet blends that provided increasing and equally spaced standardized ileal digestible Lys during late gestation

	Diet^1^							SEM^2^	*P*-Value^3^	
	70	85	100	115	130	145	160		Linear	Quadratic
Number of sows or litters^4^	15	19	20	19	20	17	18			
Lactation ADFI, kg^5^										
Week 1	3.26	3.26	3.22	3.07	3.05	3.00	3.11	0.13	0.091	0.518
Week 2	5.67	5.40	5.67	5.49	5.45	5.64	5.66	0.17	0.804	0.370
Week 3	6.79	6.57	6.63	6.53	6.73	6.74	6.72	0.17	0.786	0.288
Average	5.18	4.94	5.15	4.89	4.88	5.00	5.09	0.13	0.535	0.142
Total feed intake, kg	111.2	101.0	108.6	98.0	101.8	102.4	104.0	3.8	0.121	0.067
Estimated milk yield, kg/d^6^	10.9	10.3	11.3	11.2	11.2	11.4	11.2	0.4	0.049	0.582
Piglet body weight, kg										
Day 0	1.42	1.43	1.40	1.55	1.49	1.52	1.44	0.05	0.162	0.119
Day 7	2.93	2.74	2.88	2.96	2.96	2.87	2.82	0.10	0.974	0.447
Day 14	4.79	4.58	4.73	4.87	4.84	4.79	4.73	0.15	0.567	0.606
Wean^7^	6.60	6.14	6.58	6.56	6.50	6.54	6.39	0.16	0.916	0.669
Litter average daily gain, kg										
Week 1	2.78	2.49	2.78	2.79	2.88	2.70	2.78	1.21	0.329	0.764
Week 2	3.34	3.22	3.44	3.43	3.45	3.56	3.53	1.52	0.050	0.978
Week 3	3.19	3.20	3.51	3.34	3.33	3.57	3.37	1.43	0.068	0.330
Overall	3.19	3.00	3.31	3.27	3.27	3.33	3.28	1.13	0.057	0.561

^1^Standardized ileal digestible Lys level in the diet as a percent of estimated requirements for late gestating primiparous sows according to the NRC (2012).

^2^Maximum value for the standard error of the mean.

^3^Probability values for linear and quadratic contrasts.

^4^Overall, 24 animals were removed from the study (7, 2, 3, 3, 1, 4, and 4 sows from the 70%, 85%, 100%, 115%, 130%, 145%, and 160% treatments, respectively) for various reasons including abortion, lameness, farrowing difficulties, and savaging of piglets. Data from sows that were removed before or during lactation were not included in statistical analyses of lactation outcomes, including litter size at weaning.

^5^As-fed average daily feed intake.

^6^Milk yield was calculated using litter growth rate and litter size (NRC, 2012; Equation 8-71 and 8-72).

^7^Weaning occurred on day 20 ± 1 of lactation.

The dry matter and crude protein (as-is) contents of colostrum decreased with increasing SID Lys intake in late gestation (linear; *P *< 0.01), but crude fat and lactose contents of colostrum were not influenced by SID Lys intake in late gestation (Table 8). On day 18 of lactation, milk composition was not impacted by late-gestation SID Lys intake.

**Table 8.** Colostrum and milk composition for primiparous sows after receiving diet blends that provided increasing and equally spaced standardized ileal digestible Lys during late gestation

	Diet^1^							SEM^2^	*P*-value^3^	
	70	85	100	115	130	145	160		Linear	Quadratic
**Colostrum**										
Number of sows^4^	14	14	14	17	14	13	15			
Dry matter, %	25.1	24.0	22.2	23.8	23.8	22.5	21.8	0.9	0.007	0.888
Crude protein, %	8.5	7.8	7.1	7.8	6.9	6.9	7.0	0.6	0.037	0.454
Crude fat, %	11.8	11.1	10.2	11.2	12.3	10.9	10.0	0.8	0.246	0.582
Lactose, %	3.1	3.1	3.2	3.1	3.1	3.1	3.3	0.1	0.140	0.387
**Milk**										
Number of sows^4^	15	15	14	13	15	15	13			
Dry matter, %	20.3	20.3	20.4	20.0	20.2	19.9	20.0	0.3	0.203	0.920
Crude protein, %	5.5	5.5	5.6	5.7	5.6	5.6	5.5	0.1	0.800	0.113
Crude fat, %	8.8	8.9	9.0	8.5	8.7	8.4	8.7	0.4	0.318	0.831
Lactose, %	4.6	4.5	4.4	4.5	4.5	4.6	4.5	0.1	0.806	0.184

^1^Standardized ileal digestible Lys level in the diet as a percent of estimated requirements for late gestating primiparous sows according to the NRC (2012).

^2^Maximum value for the standard error of the mean.

^3^Probability values for linear and quadratic contrasts.

^4^Colostrum and milk were collected from representative subsets of sows, depending on farrowing time and whether personnel were available to collect colostrum and milk samples.

## Discussion

The objective of the current study was to determine the level of dietary SID Lys (protein) in late gestation that maximized whole-body nitrogen retention, as well as piglet birth weight and milk production in the following lactation. Based on the nitrogen balance results, the SID Lys necessary to maximize whole-body N retention (protein deposition) in late gestation was 22 g/d, or 15% above currently suggested requirements, with a comparable SID Lys intake in late gestation also necessary to maximize piglet birth weight. Other research groups have suggested SID Lys requirements similar to our findings to maximize whole-body protein deposition in late gestation (e.g., Ramirez-Camba et al., 2020; Johannsen et al., 2024). Since protein deposition in the fetal pool contributes approximately 50% of whole-body protein deposition in late gestation (NRC, 2012), it is not surprising that comparable SID Lys intakes maximized both outcomes (e.g., Dourmad and Étienne, 2002). By using the gestating sow model of the NRC (2012) to predict protein deposition in the pregnancy-associated and maternal pools (measured whole-body N retention × 6.25—predicted pregnancy-associated protein deposition based on observed litter size and piglet birth weights for individual sows), it was observed that protein deposition in the pregnancy-associated pool only partially accounted for the increase in whole-body protein deposition in response to SID Lys intake in late gestation. Indeed, predicted maternal protein deposition was also shown to increase (linear; *P *< 0.001; NRC, 2012; data not shown) with increasing SID Lys intake. Moreover, fasted plasma concentrations of nonessential AA decreased with increasing SID Lys intake in late gestation, suggesting that maternal protein deposition was sacrificed to meet the demands for fetal tissues when SID Lys intakes were low (Mejia-Guadarrama et al., 2002). Late gestation diets that support protein deposition in the maternal body are especially important for primiparous sows that are still growing and have nutrient and energy requirements for fetal growth and mammary development (NRC, 2012). It is noted, however, that backfat gain in late gestation was less as SID Lys intake increased in late gestation. Thus, either Lys limited whole-body (maternal + pregnancy-associated) protein deposition at lower intakes, leaving more energy for lipid deposition, or at higher Lys intakes, additional energy was required to excrete excess nitrogen (Chen et al., 1999; Johannsen et al., 2024). In addition, greater AA reservoirs in maternal tissues can be used to support increased milk production in the subsequent lactation (Cools et al., 2014; Costermans et al., 2020). In fact, lactating sows can only mobilize between 9 and 12% of body protein without negatively affecting current and future performance (Clowes et al., 2003). In the current study, sow BW loss in lactation tended to increase with increasing SID Lys intake in late gestation, while loss in backfat depth during lactation was not influenced by gestation feeding, indicating that a greater amount of maternal protein was mobilized as a source of AA, energy, or both, to support higher levels of milk production. Moreover, it has been previously shown that backfat depth less than 16 mm on day 110 of gestation negatively affected mammary development in gilts (Farmer et al., 2016). Since sows were lean in the present study (average of 13.0 mm backfat depth on day 110 of gestation), mammary development may have been impeded, while the amount of fat tissue available for mobilization was likely limited, thus additional maternal protein retention prior to farrowing provided the added benefit of greater potential energy stored in the maternal body available to support milk production.

In the current study, fostering of piglets occurred irrespective of dietary treatment and the effect of birth weight was removed after litter standardization, and no differences were observed for piglet BW at weaning. However, estimated milk yield was improved with increasing SID Lys intake in late gestation due to the cumulative effect of litter size and litter ADG. The SID Lys intake in late gestation that maximized subsequent milk yield was 22.7 g/d, though this was considered similar to the SID Lys intakes that maximized N retention in late gestation and piglet birth weights since the standard errors of the respective breakpoints overlap. As in the fetal pool, protein deposition in the mammary gland increases exponentially after day 90 of gestation (NRC, 2012), and the mammary development that occurs in late gestation can directly affect subsequent milk production capacity (reviewed by Farmer and Hurley, 2015). Had piglets only been fostered within their respective treatments, a greater difference in milk yield may have been observed, as larger birthweight piglets have greater sucking intensity and can stimulate increased milk yield (King et al., 1997). The objective of this study, however, was to look at differences in milk production relative to late-gestation dietary treatment, so fostering occurred to standardize suckling intensity. In previous work, it was demonstrated that providing SID Lys 40% above estimated requirements for primiparous sows in late gestation improved mammary development by a corresponding 44% by day 110 of gestation (Farmer et al., 2022). In the current study, however, it appeared that feeding SID Lys 40% above estimated requirements was not needed to elicit an improvement in subsequent milk yield, providing an avenue for more precise feeding strategies for late-gestating primiparous sows. It cannot be discerned whether greater protein deposition in the maternal pool, greater mammary development by the end of gestation, or a combination of the two were responsible for the improvement in estimated milk yield in the subsequent lactation with increasing SID Lys intake in late gestation. Regardless, using a phase feeding strategy to increase SID Lys (protein) intake after day 90 of gestation for primiparous sows is an attractive strategy to enhance subsequent lactation performance.

In the current study, litter ADG in the first week was not influenced by late gestation SID Lys feeding level, but in weeks 2 and 3, litter ADG tended to increase with SID Lys intake in late gestation. In the first week of lactation, milk production capacity does not limit piglet growth (Chisoro et al., 2023); thus, a lack of difference in litter ADG was not surprising, despite the lower colostral protein content with increasing Lys intake in late gestation. Conversely, in weeks 2 and 3 of lactation, litter ADG tended to increase with increasing SID Lys intake in late gestation, which is noteworthy since milk production limits piglet growth typically beyond 10 d of lactation (Chisoro et al., 2023). Therefore, providing additional SID Lys in late gestation presents an avenue for enhanced milk production later into lactation for the primiparous sow.

## Conclusion

In conclusion, in order to maximize whole-body nitrogen retention of gilts in late gestation and piglet birth weight, the intake of SID Lys in late gestation should be increased by 15% above current recommendations. To maximize subsequent milk production of primiparous sows, SID Lys intake in late gestation should be increased by 19% above current recommendations. Therefore, feeding programs for late-gestating primiparous sows should be updated in order to improve performance in the subsequent lactation. Future research should examine whether maximizing late-gestation mammary development for primiparous sows will also positively influence lifetime milk production capacity.

## Abbreviations

AA: amino acid
ADFI: average daily feed intake
ADG: average daily gain
BLL: broken line linear
N: nitrogen;
P: probability
SID: standardized ileal digestibility
